# Supplementary material for: Cytokinin regulates vegetative phase change in Arabidopsis thaliana through the miR172/TOE1-TOE2 module
Source: Nat Commun. 2021 Oct 5;12:5816. doi: 10.1038/s41467-021-26088-z (PMC8492644; doi:10.1038/s41467-021-26088-z)
Supplement: Supplementary file 1 — Supplementary Information [file 41467_2021_26088_MOESM1_ESM.pdf]

SUPPLEMENTARY INFORMATION

Cytokinin regulates vegetative phase change in Arabidopsis through the miR172/TOE1-TOE2 module

Sören Werner[1], Isabel Bartrina[1,2] & Thomas Schmülling[1,*]

[1]Institute of Biology/Applied Genetics, Dahlem Centre of Plant Sciences (DCPS), Freie Universität Berlin, Albrecht-Thaer-Weg 6, 14195 Berlin, Germany

[2]Present address: Institute of Biology, University of Graz, Schubertstrasse 51, 8010 Graz, Austria

*Corresponding author, email: t.schmuelling@fu-berlin.de

List of supplementary material

Supplementary Figures 1-11

Supplementary Tables 1-5

Supplementary References

Supplementary Figures

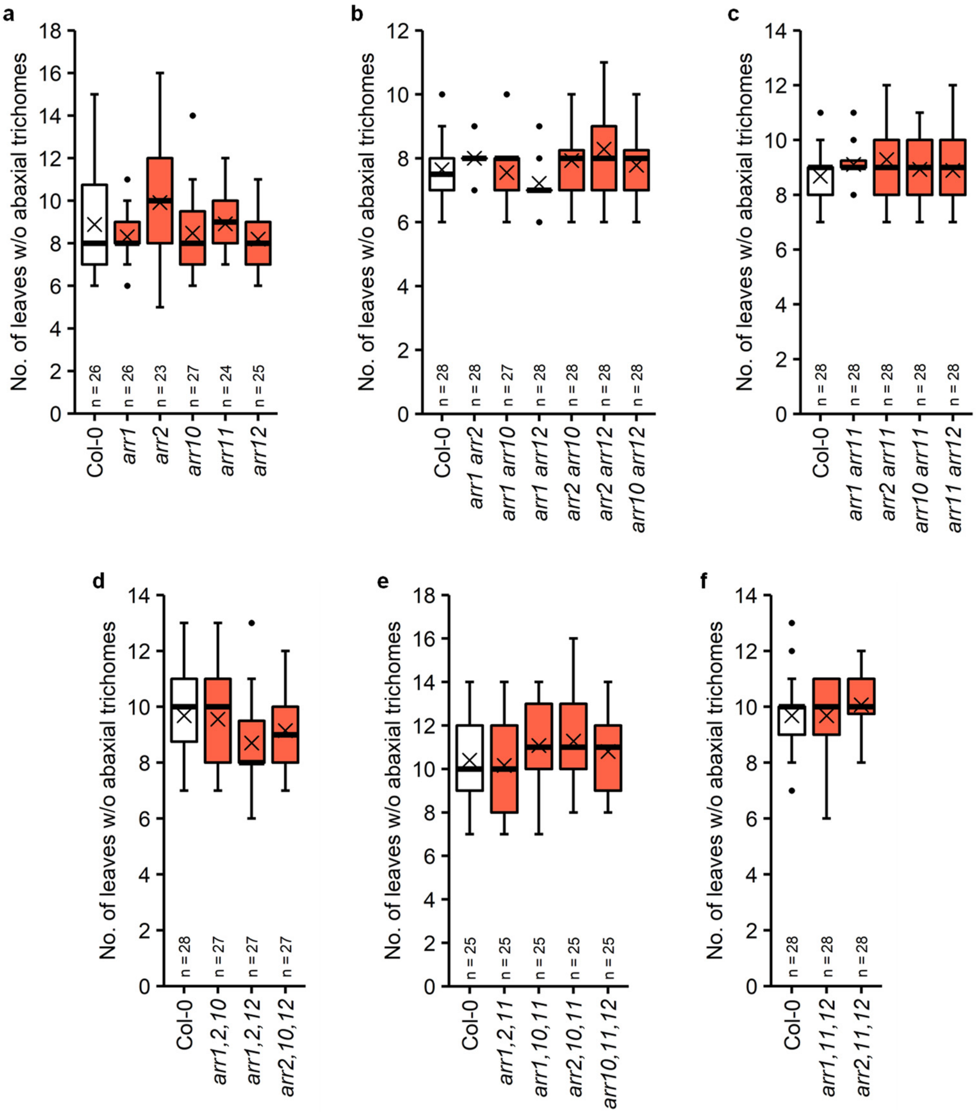

Supplementary Figure 1. Number of juvenile leaves in type-B *ARR* mutants grown in SD. Number of leaves without abaxial trichomes in type-B *ARR* single (**a**), double (**b-c**) and triple mutants (**d-f**) grown in SD. In box plots, the center line represents the median value and the boundaries indicate the 25th percentile (upper) and the 75th percentile (lower). The X marks the mean value. Whiskers extend to the largest and smallest value, excluding outliers which are shown as dots. No statistically significant differences were observed compared to the wild type of the respective experiment, as calculated by Kruskal-Wallis test ($p < 0.05$). Exact p-values are included in the Source Data files.

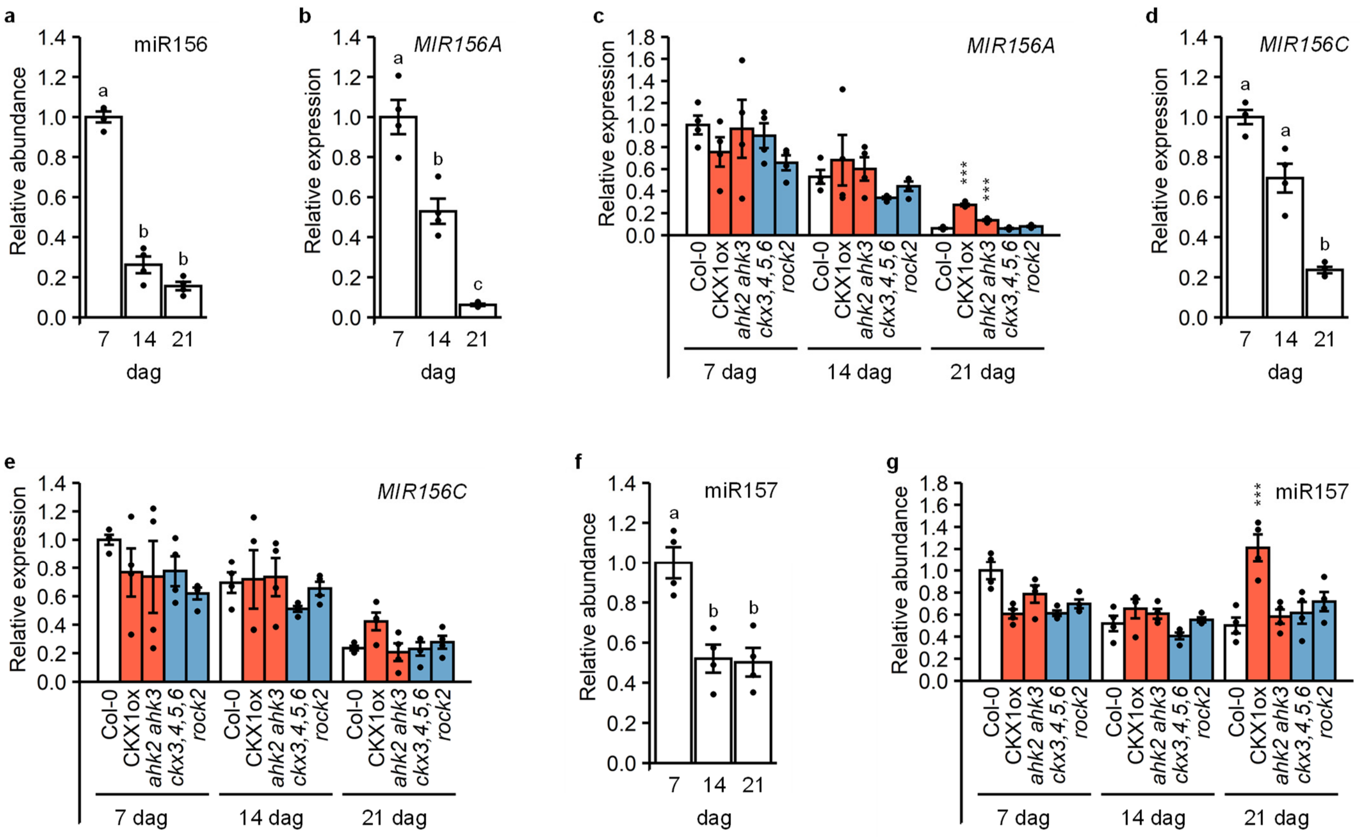

Supplementary Figure 2. *MIR156* gene expression and miR156/miR157 abundance in whole shoots of genotypes with an altered CK status compared to the wild type. Transcript levels were determined by qRT-PCR. Data were normalized to *TAFII15* (**a, f, g**) or *TAFII15* and *PP2AA2* (**b-e**). Data displayed are expressed as mean ± SEM (n = 4 biological replicates). Dots indicate each single biological replicate. Data presented in **a** (refer to Fig. 3a), **b**, **d** and **f** (refer to **c**, **e** and **g**) show the transcript level changes of the wild type over time. Letters in **a**, **b**, **d** and **f** indicate significant differences of the wild type between the different time points, as calculated by one-way ANOVA, post-hoc Tukey´s test ($p < 0.05$). Asterisks in **c**, **e** and **g** indicate significant differences compared to the wild type of the respective time point, as calculated by one-way ANOVA, post-hoc Dunnett's test (* $p < 0.05$; ** $p < 0.01$; *** $p < 0.001$). Exact p-values are included in the Source Data files.

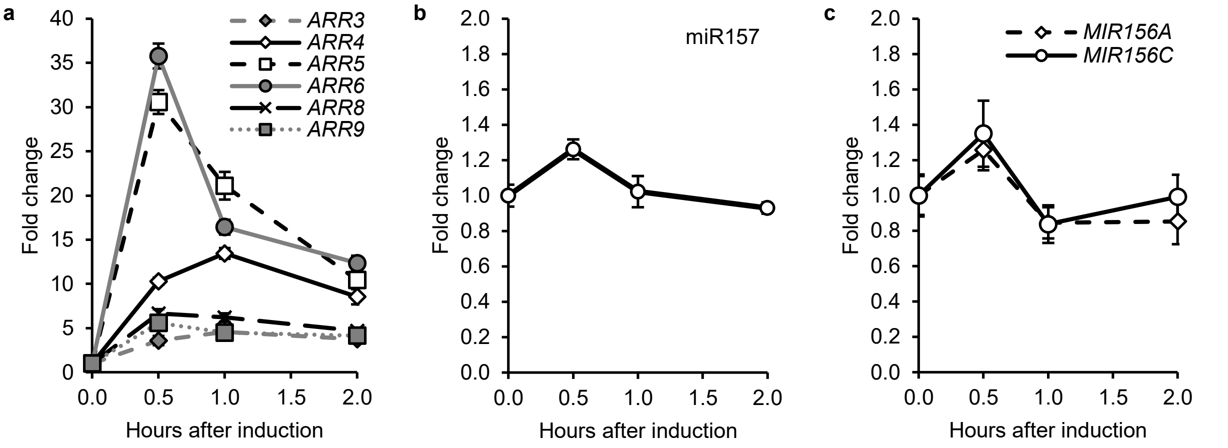

Supplementary Figure 3. Type-A *ARR* and *MIR156* and miR157 expression kinetics in wild-type seedlings after treatment with 1 µM BA. Expression kinetics were measured in 10-d-old SD-grown wild-type seedlings. Transcript levels were determined by qRT-PCR. Data were normalized to *TAFII15* and *PP2AA2* (**a, c**) or *TAFII15* (**b**). Data displayed are expressed as mean ± SEM (n = 6 biological replicates). Statistical analyses were performed using one-way ANOVA, post-hoc Dunnett's test. All *ARR* gene transcript abundances after CK application are significantly different compared to time point 0 ($p < 0.05$). No statistically significant differences in expression were observed for miR157, *MIR156A* and *MIR156C*, comparing each time point with time point 0 ($p < 0.05$). Exact p-values are included in the Source Data files.

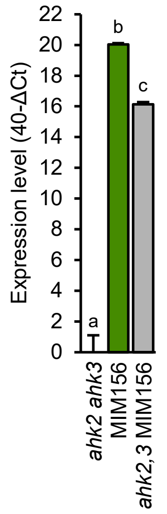

Supplementary Figure 4. *MIM156* transgene expression in *ahk2,3* MIM156. Transcript levels in 10-d-old LD-grown seedlings were determined by qRT-PCR. Data were normalized to *TAFII15*. Data displayed are expressed as mean ± SEM (n = 3 biological replicates). Letters indicate significant differences between the genotypes, as calculated by one-way ANOVA, post-hoc Tukey's test ($p < 0.05$). Exact p-values are included in the Source Data files.

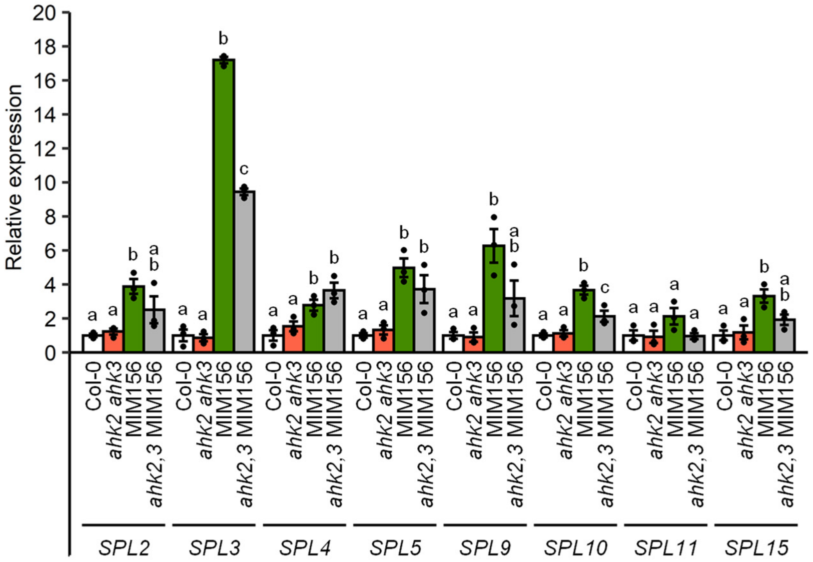

Supplementary Figure 5. *SPL* gene expression in *ahk2,3* MIM156 plants. *SPL* expression levels in 11-d-old SD-grown seedlings (n = 3 biological replicates). Transcript levels were determined by qRT-PCR. Data were normalized to *TAFII15* and *PP2AA2*. Data displayed are expressed as mean ± SEM. Dots indicate each single biological replicate. Letters indicate significant differences between the genotypes, as calculated by one-way ANOVA, post-hoc Tukey's test ($p < 0.05$). Exact p-values are included in the Source Data files.

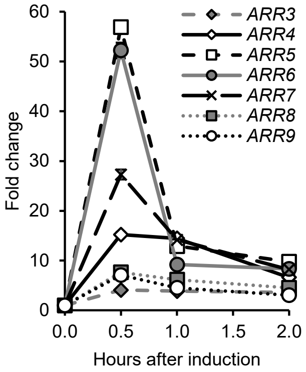

Supplementary Figure 6. Upregulation of A-type *ARR* genes in MIM156 as a cytokinin induction control. Type-B *ARR* expression kinetics in 10-d-old SD-grown MIM156 seedlings after treatment with 1 µM BA. Transcript levels were determined by qRT-PCR. Data were normalized to *TAFII15* and *PP2AA2*. Data displayed are expressed as mean ± SEM (n = 6 biological replicates). All *ARR* gene transcript abundances after CK application are significantly different compared to time point 0, as calculated by one-way ANOVA, post-hoc Dunnett's test ($p < 0.05$). Exact p-values are included in the Source Data files.

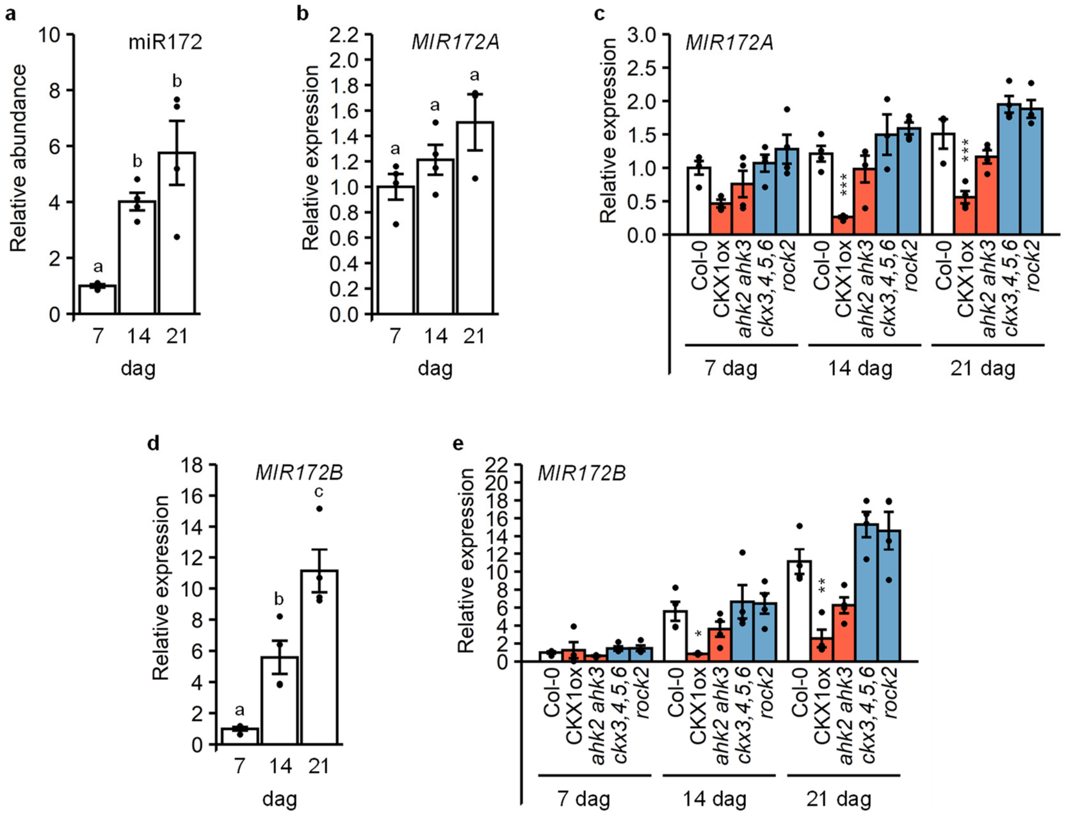

Supplementary Figure 7. MiR172 abundance and *MIR172* gene expression in whole shoots of genotypes with an altered CK status compared to the wild type. Transcript levels were determined by qRT-PCR. Data were normalized to *TAFII15* (**a**) or *TAFII15* and *PP2AA2* (**b-e**). Data displayed are expressed as mean ± SEM (n = 4 biological replicates). Dots indicate each single biological replicate. Data presented in **a** (refer to Fig. 5a), **b** and **d** (refer to **c** and **e**) show the transcript level changes of the wild type over time. Asterisks indicate significant differences compared to the wild type of the respective time point, as calculated by one-way ANOVA, post-hoc Dunnett's test (* $p < 0.05$; ** $p < 0.01$; *** $p < 0.001$). Letters indicate significant differences of the wild type between the different time points, as calculated by one-way ANOVA, post-hoc Tukey´s test ($p < 0.05$). Exact p-values are included in the Source Data files.

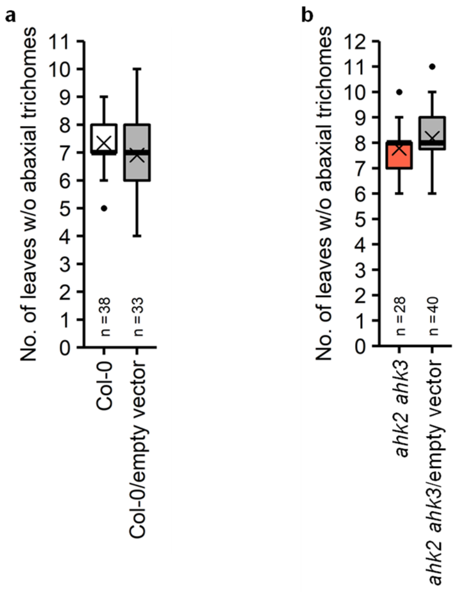

Supplementary Figure 8. Empty vector control for the *ahk2,3* MIR172Box experiment. a, b Number of leaves without abaxial trichomes in Col-0 and *ahk2-5 ahk3-7* T1 plants transformed with the empty vector (pK7m24GW) compared to untransformed Col-0 and *ahk2-5 ahk3-7* grown in SD. In box plots, the center line represents the median value and the boundaries indicate the 25th percentile (upper) and the 75th percentile (lower). The X marks the mean value. Whiskers extend to the largest and smallest value, excluding outliers which are shown as dots. No significant differences were observed compared to the respective control, as calculated by Mann-Whitney test ($p < 0.05$). Exact p-values are included in the Source Data files.

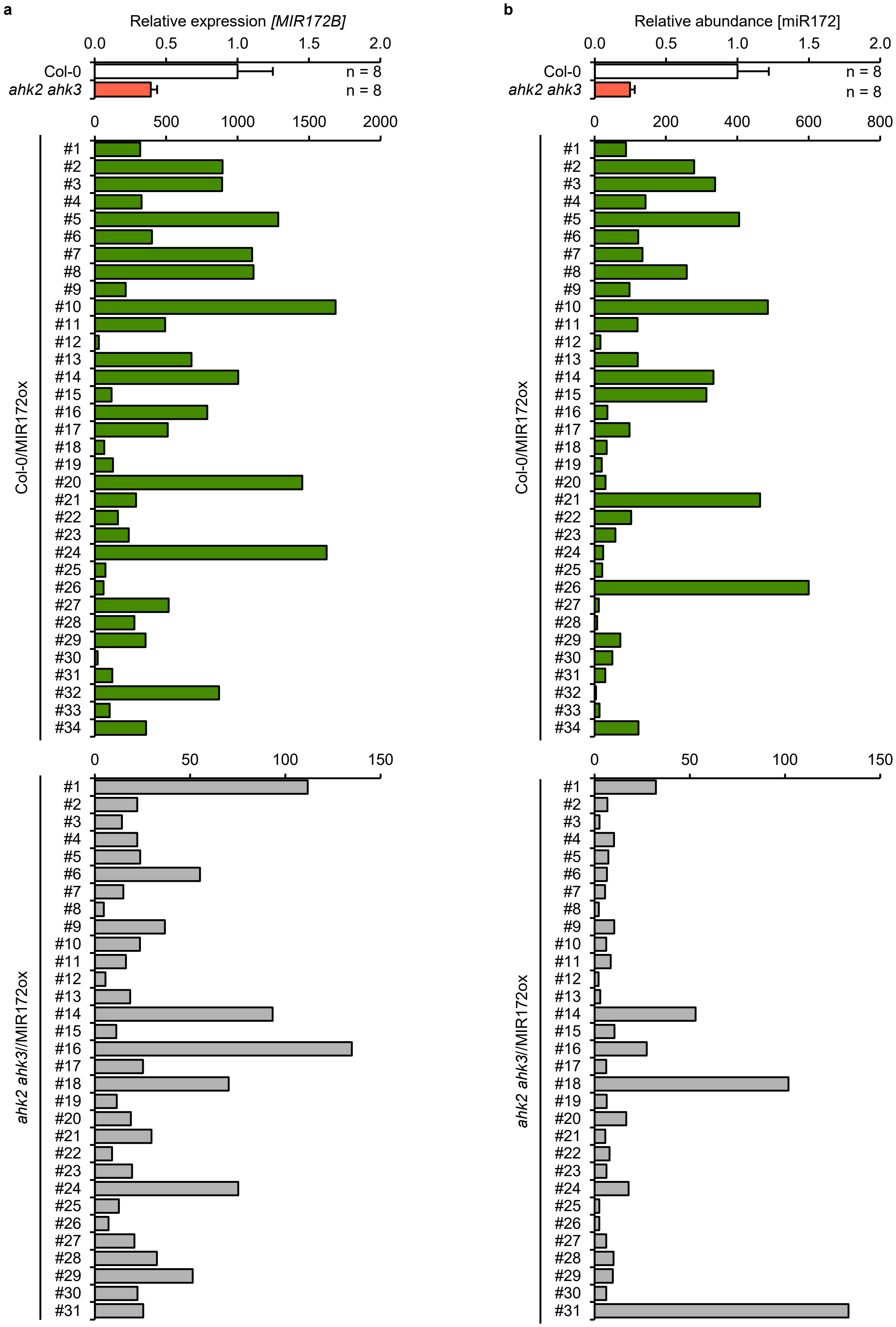

a
Relative expression [MIR172B]
0.0
0.5
1.0
1.5
2.0
Col-0
ahk2 ahk3
n = 8
n = 8
0
500
1000
1500
2000
Col-0/MIR172ox
0
50
100
150
ahk2 ahk3/MIR172ox
b
Relative abundance [miR172]
0.0
0.5
1.0
1.5
2.0
Col-0
ahk2 ahk3
n = 8
n = 8
0
200
400
600
800
Col-0/MIR172ox
0
50
100
150
ahk2 ahk3/MIR172ox
#1
#2
#3
#4
#5
#6
#7
#8
#9
#10
#11
#12
#13
#14
#15
#16
#17
#18
#19
#20
#21
#22
#23
#24
#25
#26
#27
#28
#29
#30
#31
#32
#33
#34

Supplementary Figure 9. *MIR172B* expression and miR172 abundance in *ahk2,3* MIR172Box lines. *MIR172B* transcript levels (**a**) and miR172 abundance **(b)** in leaves of untransformed wild type Col-0 and *ahk2-5 ahk3-7* (upper graphs) and in leaves of individual independent primary transformants (T1 plants) harboring the *35S:MIR172* transgene (lower graphs). Plants were grown in SD and were 42 days old when leaf material was harvested. Only T1 plants with at least two-fold increase of *MIR172B* and miR172 levels compared to their respective control are shown here and were chosen for the analysis shown in Fig. 5d. Please note the different scales in the different graphs. Expression levels were determined by qRT-PCR. Data were normalized to *TAFII15* and *PP2AA2* (**a**) or *TAFII15* (**b**).

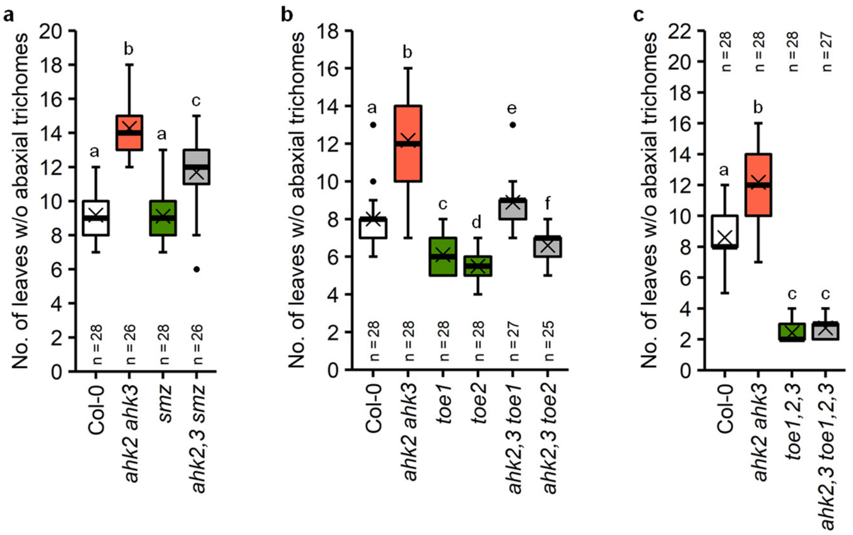

Supplementary Figure 10. Number of juvenile leaves in hybrid plants of *ahk2 ahk3* and *AP2-like* gene mutants. Number of leaves without abaxial trichomes in *ahk2,3 smz* (**a**), *ahk2,3 toe1*, *ahk2,3 toe2* (**b**) and *ahk2,3 toe1,2,3* (**c**) plants grown in SD. In box plots, the center line represents the median value and the boundaries indicate the 25th percentile (upper) and the 75th percentile (lower). The X marks the mean value. Whiskers extend to the largest and smallest value, excluding outliers which are shown as dots. Letters indicate significant differences between the genotypes, as calculated by Kruskal-Wallis test ($q < 0.05$). Exact q-values are included in the Source Data files.

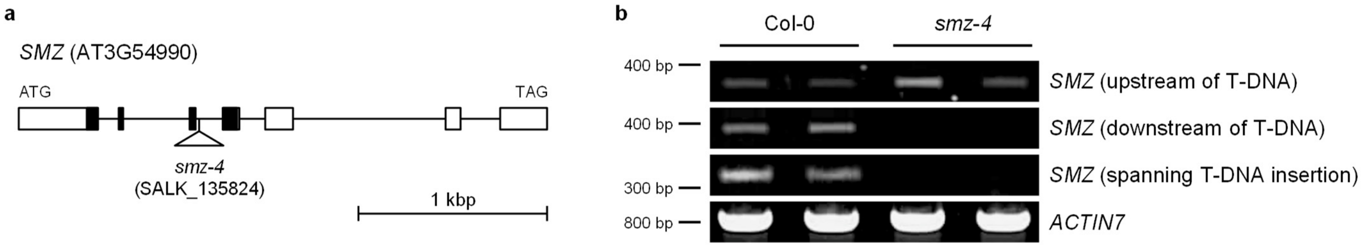

Supplementary Figure 11. Confirmation of *SMZ* knockout mutation in the *smz-4* mutant. a Schematic presentation of *SMZ* gene structure. Boxes indicate exons (the black boxes represent the coding region of the AP2/ERF DNA-binding domain), thin lines represent introns. The T-DNA is located in the third intron at bp 1035. **b** Semi-quantitative RT-PCR using primers up- or downstream of the T-DNA insertion or spanning the insertion site. No *SMZ* full-length transcript is detectable in the mutant. The experiment was performed twice with similar results. Primers used are listed in Supplementary Table 2.

Supplementary Tables

Supplementary Table 1. Mutants and transgenic lines used in this study.

**Genotype**	**Named in this study**	**Reference**
arr1-3	*arr1*	Mason et al., 2005[1]
arr2-GK	*arr2*	Nitschke et al., 2016[2]
arr10-5	*arr10*	Argyros et al., 2008[3]
arr11-3	*arr11*	Mason et al., 2005[1]
arr12-1	*arr12*	Mason et al., 2005[1]
arr1-3 arr2-GK	*arr1 arr2*	this work
arr1-3 arr10-5	*arr1 arr10*	Argyros et al., 2008[3]
arr1-3 arr11-3	*arr1 arr11*	this work
arr1-3 arr12-1	*arr1 arr12*	Mason et al., 2005[1]
arr2-GK arr10-5	*arr2 arr10*	Frank et al., 2020[4]
arr2-GK arr11-3	*arr2 arr11*	this work
arr2-GK arr12-1	*arr2 arr12*	Frank et al., 2020[4]
arr10-5 arr11-3	*arr10 arr11*	this work
arr10-5 arr12-1	*arr10 arr12*	Argyros et al., 2008[3]
arr11-3 arr12-1	*arr10 arr12*	this work
arr1-3 arr2-GK arr10-5	*arr1,2,10*	this work
arr1-3 arr2-GK arr11-3	*arr1,2,11*	this work
arr1-3 arr2-GK arr12-1	*arr1,2,12*	this work
arr1-3 arr10-5 arr11-3	*arr1,10,11*	this work
arr1-3 arr10-5 arr12-1	*arr1,10,12*	Mason et al., 2005[1]
arr1-3 arr11-3 arr12-1	*arr1,11,12*	this work
arr2-GK arr10-5 arr11-3	*arr2,10,11*	this work
arr2-GK arr10-5 arr12-1	*arr2,10,12*	Frank et al., 2020[4]
arr2-GK arr11-3 arr12-1	*arr2,11,12*	this work
arr10-5 arr11-3 arr12-1	*arr10,11,12*	this work
ahk2-2tk cre1-12	*ahk2 cre1*	Higuchi et al., 2004[5]
ahk3-3 cre1-12	*ahk3 cre1*	Higuchi et al., 2004[5]
ahk2-2tk ahk3-3	*ahk2 ahk3*	Higuchi et al., 2004[5]
ahk2-5 ahk3-7	*ahk2 ahk3*	Riefler et al., 2006[6]
rock2	*rock2*	Bartrina et al., 2017[7]
rock3	*rock3*	Bartrina et al., 2017[7]
abcg14-2	*abcg14*	Zhang et al., 2014[8]
ahp2 ahp3 ahp5-2	*ahp2,3,5*	Hutchison et al., 2006[9]
arr3,4,5,6,8,9	*arr3,4,5,6,8,9*	To et al., 2004[10]
ckx3-1 ckx5-1	*ckx3 ckx5*	Bartrina et al., 2011[11]
ckx3-1 ckx4-1 ckx5-1 ckx6-2	*ckx3,4,5,6*	this work
cyp735a1-2 cyp735a2-2	*cypDM*	Kiba et al., 2013[12]
ipt2-1 ipt9-1	*ipt2 ipt9*	Miyawaki et al., 2006[13]
ipt3-2 ipt5-2 ipt7-1	*ipt3,5,7*	Miyawaki et al., 2006[13]
log4-3	*log4*	Kuroha et al., 2009[14]
log4-3 log7-1	*log4 log7*	Kuroha et al., 2009[14]
log3-1 log4-3 log7-1	*log3,4,7*	Kuroha et al., 2009[14]
p35S:CKX1	CKX1ox	Werner et al., 2003[15]
p35S:MIM156	MIM156	Franco-Zorilla et al., 2007[16]
ahk2-2tk ahk3-3 p35S:MIM156	*ahk2,3* MIM156	this work
spl9-4 spl15-1	*spl9 spl15*	Wu et al., 2009[17]
rock2 spl9-4 spl15-1	*rock2 spl9,15*	this work
p35S:MIR156B	MIR156ox	Wang et al., 2009[18]

Supplementary Table 1. Continuation.

rock2 p35S:MIR156B	*rock2* MIR156ox	this work
p35S:MIR172B	MIR172ox	this work
ahk2-5 ahk3-7 p35S:MIR172B	*ahk2,3* MIR172ox	this work
smz-4 (SALK_135824C)	*smz*	this work
snz-1	*snz*	Mathieu et al., 2009[19]
smz-4 snz-1	*smz snz*	this work
ahk2-2tk ahk3-3 smz-4	*ahk2,3 smz*	this work
ahk2-2tk ahk3-3 smz-4 snz-1	*ahk2,3 smz snz*	this work
toe1-2 toe2-1	*toe1 toe2*	Aukerman & Sakai, 2003[20]
toe3-2	*toe3*	Jung et al., 2014[21]
toe1-2 toe2-1 toe3-2	*toe1,2,3*	this work
ahk2-2tk ahk3-3 toe1-2 toe2-1	*ahk2,3 toe1,2*	this work
ahk2-2tk ahk3-3 toe1-2 toe2-1 toe3-2	*ahk2,3 toe1,2,3*	this work
rock2 toe1-2 toe2-1	*rock2 toe1,2*	this work

Supplementary Table 2. Primers for the analysis of *SMZ* gene knockout in the *smz-4* mutant.

Gene/gene region	Primers	Sequences (5' → 3')
ACTIN7	052_ACTIN7_fw	TACAACGAGCTTCGTGTTGC
	053_ACTIN7_rv	TCCACATCTGTTGGAAGGTG
SMZ 5' end	507_SMZ-5'_fw	GCAAGTTTATTTGGGCGGG
	292_SMZ-5'_rv	CCTTGGTCCTCTTCTGCTCTTC
SMZ T-DNA spanning	509_SMZ-T-DNA_fw	AGGCTTGGCTCTTCAAAAATG
	510_SMZ-T-DNA_rv	ATGGCTCCTTCTCCCTTTCC
SMZ 3' end	529_SMZ-3'_fw	ATGTTGGATCTTAACCTAAAGA
	530_SMZ-3'_rv	CTATGGATCAAAACAATTGG

Supplementary Table 3. Gene-specific primers used for genotyping in this study.

Locus	Allele	Primer pair	Sequences (5' → 3')	Fragment size
ARR1 (AT3G16857)	WT	309_ARR1-arr1-3_fw<br>310_ARR1-arr1-3_rv	CTTCAAGCACTAGCCGTCACAGGTCAGTT<br>AATGTTATCGATGGAGTATGCGTCAAAGT	1306 bp
	arr1-3	309_ARR1-arr1-3_fw<br>315_LBa1	CTTCAAGCACTAGCCGTCACAGGTCAGTT<br>TGGTTCACGTAGTGGGCCATCG	953 bp
ARR2 (AT4G16110)	WT	538_ARR2-arr2-GK_fw<br>539_ARR2-arr2-GK_rv	ATGGTAAATCCGGGTCAC<br>ACATTCCACTCGTTACGC	1017 bp
	arr2-GK	540_GABI-LB<br>539_ARR2-arr2-GK_rv	ATATTGACCATCATACTCATTGC<br>ACATTCCACTCGTTACGC	~ 1100 bp
ARR10 (AT4G31920)	WT	311_ARR10-arr10-5_fw<br>312_ARR10-arr10-5_rv	CATTGGAGTTGTTGAGGGAGA<br>CGATGATGAGACTGGTTGGA	1075 bp
	arr10-5	311_ARR10-arr10-5_fw<br>315_LBa1	CATTGGAGTTGTTGAGGGAGA<br>TGGTTCACGTAGTGGGCCATCG	1230 bp
ARR11 (AT1G67710)	WT	589_ARR11-arr11-3_fw<br>590_ARR11-arr11-3_rv	GAAGCCGATAAGAATGAAGGAG<br>GCCAAGTCAAGTCTAAGTCAGTG	1255 bp
	arr11-3	589_ARR11-arr11-3_fw<br>400_LBb1.3-SALK	GAAGCCGATAAGAATGAAGGAG<br>ATTTTGCCGATTTCGGAAC	882 bp
ARR12 (AT2G25180)	WT	313_ARR12-arr12-1_fw<br>314_ARR12-arr12-1_rv	TAACAACGACGAACCAAGCA<br>TTGGCAGAGTCACAGAATGG	1654 bp
	arr12-1	313_ARR12-arr12-1_fw<br>315_LBa1	TAACAACGACGAACCAAGCA<br>TGGTTCACGTAGTGGGCCATCG	962 bp
AHK2 (AT5G35750)	WT *(ahk2-2tk)*	446_AHK2-ahk2-2tk-2_fw<br>447_AHK2-ahk2-2tk-2_rv	TGCCTTGTCTATTCTTGTATCT<br>TAGGTTCAATTTCTTCAGTCC	719 bp
	ahk2-2tk	446_AHK2-ahk2-2tk-2_fw<br>230_T-DNA-ahk2-2tk_rv	TGCCTTGTCTATTCTTGTATCT<br>ATAACGCTGCGGACATCTAC	~ 700 bp
	WT *(ahk2-5)*	222_AHK2-ahk2-5_fw<br>223_AHK2-ahk2-5_rv	GCAAGAGGCTTTAGCTCCAA<br>TTGCCCGTAAGATGTTTTCA	772 bp
	ahk2-5	224_LB1-SAIL<br>223_AHK2-ahk2-5_rv	GCCTTTTCAGAAATGGATAAATAGCCTTGCTTCC<br>TTGCCCGTAAGATGTTTTCA	650 bp
AHK3 (AT1G27320)	WT *(ahk3-3)*	397_AHK3-ahk3-3_fw<br>449_AHK3-ahk3-3_rv	GCAAGAATCCAGGTGCTAAC<br>GCTATCAGTTACAACCCTTGC	771 bp
	ahk3-3	315_LBa1<br>449_AHK3-ahk3-3_rv	TGGTTCACGTAGTGGGCCATCG<br>GCTATCAGTTACAACCCTTGC	~ 850 bp

Supplementary Table 3. Continuation.

AHK3 (AT1G27320)	WT *(ahk3-7)*	AC_AHK3-ahk3-7_fw<br>AC_AHK3-ahk3-7_rv	CCTTGTTGCCTCTCGAACTC<br>CGCAAGCTATGGAGAAGAGG	558 bp
	ahk3-7	AC_GABI-IG1<br>AC_AHK3-ahk3-7_rv	CCCATTTGGACGTGTAGACAC<br>CGCAAGCTATGGAGAAGAGG	~ 550 bp
CRE1 (AT2G01830)	WT	213_CRE1-cre1-12_fw<br>214_CRE1-cre1-12_rv	GGAGAGCCTTCACCGGTTAGG<br>AAGCTCTTGCATTTCATGGAAATC	982 bp
	cre1-12	213_CRE1-cre1-12_fw<br>315_LBa1	GGAGAGCCTTCACCGGTTAGG<br>TGGTTCACGTAGTGGGCCATCG	~ 500 bp
ROCK2	WT	263_rock2_fw<br>264_rock2_rv	TGGCTCAGAAATTGGGGATA<br>TGATGGCTTCATATAAAATATAACCATCTA	250 + 31 bp<br>cleaved by XbaI
	rock2	263_rock2_fw<br>264_rock2_rv	TGGCTCAGAAATTGGGGATA<br>TGATGGCTTCATATAAAATATAACCATCTA	281 bp<br>not cleaved by XbaI
ROCK3	WT	265_rock3_fw<br>266_rock3_rv	TTTTGCTGATTGTTGCTTACAG<br>CCTATCAAGAGGAATTGAGTGAGA	595 bp<br>not cleaved by XapI (ApoI)
	rock3	265_rock3_fw<br>266_rock3_rv	TTTTGCTGATTGTTGCTTACAG<br>CCTATCAAGAGGAATTGAGTGAGA	459 + 136 bp<br>cleaved by XapI (ApoI)
CYP735A1 (AT5G38450)	WT	581_CYP735A1-cyp735a1_fw<br>582_CYP735A1-cyp735a1_rv	CTTGCGGTTACGTCGGCTC<br>ATCCTCATGAAACCAATGGCTTC	1357 bp
	cyp735a1-2	399_LBb1-SALK<br>582_CYP735A1-cyp735a1_rv	GCGTGGACCGCTTGCTGCAACT<br>ATCCTCATGAAACCAATGGCTTC	~ 500 bp
CYP735A2 (AT1G67110)	WT	585_CYP735A1-cyp735a2-2_fw<br>586_CYP735A1-cyp735a2-2_rv	CCAAGATGGTGTCCCTTCCG<br>CTTGGTAAAAGTGTGGCAGGAG	1518 bp
	cyp735a2-2	399_LBb1-SALK<br>586_CYP735A1-cyp735a2-2_rv	GCGTGGACCGCTTGCTGCAACT<br>CTTGGTAAAAGTGTGGCAGGAG	~ 700 bp
SPL9 (AT2G42200)	WT	420_SPL9-spl9-4_fw<br>421_SPL9-spl9-4_rv	TGGTTCCTCCACTGAGTCATC<br>GCTCATTATGACCAGCGAGTC	1021 bp
	spl9-4	401_LB1-SAIL<br>421_SPL9-spl9-4_rv	GCCTTTTCAGAAATGGATAAATAGCCTTGCTTCC<br>GCTCATTATGACCAGCGAGTC	~ 650 bp
SPL15 (AT3G57920)	WT	422_SPL15-spl15-1_fw<br>423_SPL15-spl15-1_rv	TCCACCGAGTCTTCTTCACTC<br>TGTTGGTGTCTGAAGTTGCTG	1013 bp
	spl15-1	400_LBb1.3-SALK<br>423_SPL15-spl15-1_rv	ATTTTGCCGATTTCGGAAC<br>TGTTGGTGTCTGAAGTTGCTG	~ 800 bp

Supplementary Table 3. Continuation.

TOE1 (AT2G28550)	WT	404_TOE1-toe1-2_fw<br>405_TOE1-toe1-2_rv	GAAGAGTTTGTGCATATACTGCG<br>GAAGGGAAGTGAAAGAGCCTC	569 bp
	toe1-2	400_LBb1.3-SALK<br>405_TOE1-toe1-2_rv	ATTTTGCCGATTTCGGAAC<br>GAAGGGAAGTGAAAGAGCCTC	~ 400 bp
TOE2 (AT5G60120)	WT	406_TOE2-toe2-1_fw<br>407_TOE2-toe2-1_rv	AGTTGTGCTCTACACGAACGG<br>TCCCAGCAGAAATCAGTTCAC	580 bp
	toe2-1	406_TOE2-toe2-1_fw<br>400_LBb1.3-SALK	AGTTGTGCTCTACACGAACGG<br>ATTTTGCCGATTTCGGAAC	~ 350 bp
TOE3 (AT5G67180)	WT	408_TOE3-toe3-2_fw<br>409_TOE3-toe3-2_rv	ATCCTGCACCTCTCTAATGATG<br>GACACTATTGAAACCGGACCA	385 bp
	toe3-2	401_LB1-SAIL<br>409_TOE3-toe3-2_rv	GCCTTTTCAGAAATGGATAAATAGCCTTGCTTCC<br>GACACTATTGAAACCGGACCA	~ 600 bp
SMZ (AT3G54990)	WT	291_SMZ-smz-4_fw<br>411_SMZ-smz-4_rv	GTGGTGGCTGATGCTCGTC<br>AATCATCCACGACGAAATTGATGTCTG	923 bp
	smz-4	410_SMZ-smz-4_fw2<br>400_LBb1.3-SALK	TTTGATTTGTAGATCTTCTCTGACAAC<br>ATTTTGCCGATTTCGGAAC	~ 700 bp
SNZ (AT2G39250)	WT	412_SNZ-snz-1_fw<br>413_SNZ-snz-1_rv	AGGTCCCCAACACGTTCCATT<br>ATCCAACCACTCATTTCCGGG	955 bp
	snz-1	400_LBb1.3-SALK<br>413_SNZ-snz-1_rv	ATTTTGCCGATTTCGGAAC<br>ATCCAACCACTCATTTCCGGG	~ 600 bp
LOG3 (AT2G37210)	WT	613_LOG3-log3-1_fw<br>614_LOG3-log3-1_rv	CATTCCCAAGACCCTCATGCCTAGA<br>CTAATTTTAAGTGCCAGATGTTGAT	1037 bp
	log3-1	613_LOG3-log3-1_fw<br>315_LBa1	CATTCCCAAGACCCTCATGCCTAGA<br>TGGTTCACGTAGTGGGCCATCG	~ 650 bp
LOG4 (AT3G53450)	WT	DP_LOG4-log4-3_fw<br>DP_LOG4-log4-3_rv	GGTTTGCTTTGTAATGATTTCTGGG<br>TCAGTCTTCAGAAGAGTAGTCAATC	923 bp
	log4-3	315_LBa1<br>DP_LOG4-log4-3_rv	TGGTTCACGTAGTGGGCCATCG<br>TCAGTCTTCAGAAGAGTAGTCAATC	~ 700 bp
LOG7 (AT5G06300)	WT	DP_LOG7-log7-1_fw<br>DP_LOG7-log7-1_rv	GTCATTACATGGGCTCAACTCGGTA<br>TCACAATCAGGGGTTATGTAGTCGT	449 bp
	log7-1	315_LBa1<br>DP_LOG7-log7-1_rv	TGGTTCACGTAGTGGGCCATCG<br>TCACAATCAGGGGTTATGTAGTCGT	~ 750 bp

Supplementary Table 3. Continuation.

IPT3 (AT3G63110)	WT	AC_IPT3-ipt3-2_fw<br>AC_IPT3-ipt3-2_rv	CCAACTTGTCGTATATCATTCGTACAGTG<br>TGGAGAGATTCGCCATGTGACAG	549 bp
	ipt3-2	AC_IPT3-ipt3-2_fw<br>AC_T-DNA-ipt3-2	CCAACTTGTCGTATATCATTCGTACAGTG<br>CAACACGTGGGTTAATTAAGAATTCAGTAC	~ 450 bp
IPT5 (AT5G19040)	WT	AC_IPT5-ipt5-2_fw<br>AC_IPT5-ipt5-2_rv	TGCATGACGGCTCTAAGACA<br>TCGAGCTCTGGAACTCCAAT	617 bp
	ipt5-2	315_LBa1<br>AC_IPT5-ipt5-2_rv	TGGTTCACGTAGTGGGCCATCG<br>TCGAGCTCTGGAACTCCAAT	~ 300 bp
IPT7 (AT3G23630)	WT	AC_IPT7-ipt7-1_fw<br>AC_IPT7-ipt7-1_rv	CTACCGGATCGGGTAAGTCTC<br>GCTACAAGATTCTCCCAAGCC	736 bp
	ipt7-1	AC_IPT7-ipt7-1_fw<br>315_LBa1	CTACCGGATCGGGTAAGTCTC<br>TGGTTCACGTAGTGGGCCATCG	~ 350 bp

**Locus**	**Allele**	**Reference**
ABCG14 (AT1G31770)	*abcg14-2*	Zhang et al., 2014[8]
AHP2 (AT3G29350)	*ahp2*	Hutchison et al., 2006[9]
AHP3 (AT5G39340)	*ahp3*	Hutchison et al., 2006[9]
AHP5 (AT1G03430)	*ahp5-2*	Hutchison et al., 2006[9]
ARR3 (AT1G59940)	*arr3*	To et al., 2004[10]
ARR4 (AT1G10470)	*arr4*	To et al., 2004[10]
ARR5 (AT3G48100)	*arr5*	To et al., 2004[10]
ARR6 (AT5G62920)	*arr6*	To et al., 2004[10]
ARR8 (AT2G41310)	*arr8*	To et al., 2004[10]
ARR9 (AT3G57040)	*arr9*	To et al., 2004[10]
CKX3 (AT5G56970)	*ckx3-1*	Bartrina et al., 2011[11]
CKX4 (AT4G29740)	*ckx4-1*	Bartrina et al., 2011[11]
CKX5 (AT1G75450)	*ckx5-1*	Bartrina et al., 2011[11]
CKX6 (AT3G63440)	*ckx6-2*	Bartrina et al., 2011[11]
IPT2 (AT2G27760)	*ipt2-1*	Miyawaki et al., 2006[13]
IPT9 (AT5G20040)	*ipt9-1*	Miyawaki et al., 2006[13]

Supplementary Table 4. Primers used for cloning.

DNA fragment	Primer pair	Sequences (5' → 3')
p35S	EO_p35S-F-**attB4**	**GGGGACAACTTTGTATAGAAAAGTTGTC**CTATGACCATGATTACGCC
	EO_p35S-R-**attB1r**	**GGGGACTGCTTTTTTGTACAAACTTGC**CCGTGTTCTCTCCAAATG
MIR172B	534_MIR172B-**attB1**-2_fw	**GGGGACAAGTTTGTACAAAAAAGCAGGCT**AGTCTTGTGTGCACCCATTTATGTAG
	535_MIR172B-**attB2**-2_rv	**GGGGACCACTTTGTACAAGAAAGCTGGGTA**CTCAAGTCAAGATCAAAGGCAAAATAG

Supplementary Table 5. Stem-loop and qRT primers used in this study.

Gene	Primers	Sequences (5' → 3')
**Primers used for stem-loop cDNA synthesis**		
	484_TAFII15-StLp-cDNA_rv	CTATTCGTCCCTTGTTG
	452_miR156/miR157-**StLp**	**GTCGTATCCAGTGCAGGGTCCGAGGTATTCGCACTGGATACGAC**GTGCT
	455_miR172abe-**StLp**	**GTCGTATCCAGTGCAGGGTCCGAGGTATTCGCACTGGATACGAC**ATGCA
**qRT primers used for detection of mature miRNAs**		
	453_StLp-qRT_fw	CAGTGCAGGGTCCGAGGT
	454_miR156-qRT_rv	CCGTGGTGACAGAAGAGAGTGA
	469_miR157-qRT_rv	CCGTGGTTGACAGAAGATAGAGA
	459_miR172-qRT_rv	GTCCGTGGAGAATCTTGATGATG
**qRT primers used for detection of transgene expression**		
MIM156	487_MIM156-qRT_fw	AAATGCTCACTTCTATCTTCTGTCAA
	489_MIM-qRT_rv	TCCTCACACAAAGAACACACAAC
**qRT primers used for detection of gene transcripts**		
PP2AA2 (AT3G25800)	478_PP2AA2-qRT_fw	CCATTAGATCTTGTCTCTCTGCT
	479_PP2AA2-qRT_rv	GACAAAACCCGTACCGAG
TAFII15 (AT4G31720)	339_TAFII15-qRT_fw	GAATCACGGCCAACAATC
	340_TAFII15-qRT_rv	ACTCTTAGCCAAGTAGTGCTCC
ARR3 (AT1G59940)	591_ARR3-qRT_fw	CTCGATGGAATCTTCGGA
	592_ARR3-qRT_rv	CACAAGCGAAGTTGCAGA
ARR4 (AT1G10470)	490_ARR4-qRT_fw	CCGTTGACTATCTCGCCT
	491_ARR4-qRT_rv	CGACGTCAACACGTCATC

Supplementary Table 5. Continuation.

ARR5 (AT3G48100)	593_ARR5-qRT_fw<br>594_ARR5-qRT_rv	CTACTCGCAGCTAAAACGC<br>GCCGAAAGAATCAGGACA
ARR6 (AT5G62920)	492_ARR6-qRT_fw<br>493_ARR6-qRT_rv	GAGCTCTCCGATGCAAAT<br>GAAAAAGGCCATAGGGGT
ARR7 (AT1G19050)	595_ARR7-qRT_fw<br>596_ARR7-qRT_rv	CTTGGAACCAATCTGCTCTC<br>ATCATCGACGGCAAGAAC
ARR8 (AT2G41310)	597_ARR8-qRT_fw<br>598_ARR8-qRT_rv	CAACCCGAGAAGCCACTA<br>ACGATGTTGCTGCGGTAT
ARR9 (AT3G57040)	599_ARR9-qRT_fw<br>600_ARR9-qRT_rv	GATAGAGCACGTCCTAGATTCG<br>CTGCATTCCCTACTGAAACC
MIR156A (AT2G25095)	269_MIR156A-qRT_fw<br>270_MIR156A-qRT_rv	TGGGACAAGAGAAACGCAAAG<br>TGAGCACGCAAGAGAAGCAAGT
MIR156C (AT4G31877)	365_MIR156C-qRT_fw<br>366_MIR156C-qRT_rv	TCTGCCTCCTTTCCAATCTTCT<br>TTATCACCTTTACCTTACCGACACA
MIR172A (AT2G28056)	631_MIR172A-qRT3_fw<br>632_MIR172A-qRT3_rv	GTCGTTGTTGGCTGCTGTG<br>ATAGAGAACTTTGTGGAGAGTGAATC
MIR172B (AT2G28056)	343_MIR172B-qRT2_fw<br>344_MIR172B-qRT2_rv	GTTGTTTGTAGGCGCAGCAC<br>GCAGCATCATCAAGATTCTCATATAC
SPL2 (AT1G54150)	271_SPL2-qRT_fw<br>272_SPL2-qRT_rv	CAAAACCGCAAGAGCCGAG<br>TGACAGAAGAGAGAGAGCACCATC
SPL3 (AT2G33810)	273_SPL3-qRT_fw<br>274_SPL3-qRT_rv	AGAAGGCGGAAAAGCACAAC<br>TGGAGAAACAGACAGAGACACAGAG
SPL4 (AT1G53160)	275_SPL4-qRT_fw<br>276_SPL4-qRT_rv	AAGGCATCTTCTGTCTTTCTCTC<br>CTGACCATTGATTCCTCTCCG
SPL5 (AT3G15270)	277_SPL5-qRT_fw<br>278_SPL5-qRT_rv	CTCATCATTCAAGCGACCACAG<br>TTACAGGACAGCATAGAGGGGAC
SPL6 (AT1G69170)	279_SPL6-qRT_fw<br>280_SPL6-qRT_rv	TGAGCAAAACAGCAGCAGAAC<br>CTGACAGAAGAGAGAGAGCACGAG

Supplementary Table 5. Continuation.

SPL9 (AT2G42200)	281_SPL9-qRT_fw	GCACGGCAATGGGTGAGTT
	282_SPL9-qRT_rv	CAGTTGGTATGGTGAGAAGAAGAGTC
SPL10 (AT1G27370)	283_SPL10-qRT_fw	ACCCTCTCTTTCTCTGCGTTTC
	284_SPL10-qRT_rv	GAGCATTCTCCCACACCTTTG
SPL11 (AT1G27360)	285_SPL11-qRT_fw	AGTGGGATTGGGAGCATTTG
	286_SPL11-qRT_rv	CGATACAGCAGTGTGCCAGAAG
SPL13A (AT5G50570)/ *SPL13B* (AT5G50670)	558_SPL13-qRT_fw	ATCTCGTTTGGTGGGTCGTC
	559_SPL13-qRT_rv	AATCGGCATCTGGTTGGTTC
SPL15 (AT3G57920)	287_SPL15-qRT_fw	TGGAGAAAAGAAGTTGTCGCAG
	288_SPL15-qRT_rv	GTGAAAAGAGCCGTTGTGGG

Supplementary References

1. Mason, M. G. et al. Multiple type-B response regulators mediate cytokinin signal transduction in *Arabidopsis*. *Plant Cell* **17**, 3007-3018 (2005).
2. Nitschke, S. et al. Circadian stress regimes affect the circadian clock and cause jasmonic acid-dependent cell death in cytokinin-deficient *Arabidopsis* plants. *Plant Cell* **28**, 1616-1639 (2016).
3. Argyros, R. D. et al. Type B response regulators of *Arabidopsis* play key roles in cytokinin signaling and plant development. *Plant Cell* **20**, 2102-2116 (2008).
4. Frank, M., Cortleven, A., Novák, O. & Schmülling, T. Root-derived *trans*-zeatin cytokinin protects *Arabidopsis* plants against photoperiod stress. *Plant Cell Environ* **43**, 2637-2649 (2020).
5. Higuchi, M. et al. *In planta* functions of the *Arabidopsis* cytokinin receptor family. *Proc. Natl. Acad. Sci. USA* **101**, 8821-8826 (2004).
6. Riefler, M., Novak, O., Strnad, M. & Schmülling, T. *Arabidopsis* cytokinin receptor mutants reveal functions in shoot growth, leaf senescence, seed size, germination, root development, and cytokinin metabolism. *Plant Cell* **18**, 40-54 (2006).
7. Bartrina, I. et al. Gain-of-function mutants of the cytokinin receptors AHK2 and AHK3 regulate plant organ size, flowering time and plant longevity. *Plant Physiology* **173**, 1783-1797 (2017).
8. Zhang, K. et al. *Arabidopsis* ABCG14 protein controls the acropetal translocation of root-synthesized cytokinins. *Nat. Commun.* **5**, 3274 (2014).
9. Hutchison, C. E. et al. The *Arabidopsis* histidine phosphotransfer proteins are redundant positive regulators of cytokinin signaling. *Plant Cell* **18**, 3073-3087 (2006).
10. To, J. P. C. et al. Type-A *Arabidopsis* response regulators are partially redundant negative regulators of cytokinin signaling. *Plant Cell* **16**, 658-671 (2004).
11. Bartrina, I., Otto, E., Strnad, M., Werner, T. & Schmülling, T. Cytokinin regulates the activity of reproductive meristems, flower organ size, ovule formation, and thus seed yield in *Arabidopsis thaliana*. *Plant Cell* **23**, 69-80 (2011).
12. Kiba, T., Takei, K., Kojima, M. & Sakakibara, H. Side-chain modification of cytokinins controls shoot growth in *Arabidopsis*. *Dev. Cell* **27**, 452-461 (2013).
13. Miyawaki, K. et al. Roles of *Arabidopsis* ATP/ADP isopentenyltransferases and tRNA isopentenyltransferases in cytokinin biosynthesis. *Proc. Natl. Acad. Sci. USA* **103**, 16598-16603 (2006).
14. Kuroha, T. et al. Functional analyses of LONELY GUY cytokinin-activating enzymes reveal the importance of the direct activation pathway in *Arabidopsis*. *Plant Cell* **21**, 3152-3169 (2009).

15. Werner, T. et al. Cytokinin-deficient transgenic *Arabidopsis* plants show multiple developmental alterations indicating opposite functions of cytokinins in the regulation of shoot and root meristem activity. *Plant Cell* **15**, 2532-2550 (2003).

16. Franco-Zorrilla, J. M. et al. Target mimicry provides a new mechanism for regulation of microRNA activity. *Nature Genet.* **39**, 1033-1037 (2007).

17. Wu, G. et al. The sequential action of miR156 and miR172 regulates developmental timing in *Arabidopsis*. *Cell* **138**, 750-759 (2009).

18. Wang, J. W., Czech, B. & Weigel, D. miR156-regulated SPL transcription factors define an endogenous flowering pathway in *Arabidopsis thaliana*. *Cell* **138**, 738-749 (2009).

19. Mathieu, J., Yant, L. J., Mürdter, F., Küttner, F. & Schmid, M. Repression of flowering by the miR172 target *SMZ*. *PLoS Biol.* **7**, e1000148 (2009).

20. Aukerman, M. J. & Sakai, H. Regulation of flowering time and floral organ identity by a microRNA and its *APETALA2*-like target genes. *Plant Cell* **15**, 2730-2741 (2003).

21. Jung, J. H., Lee, S., Yun, J., Lee, M. & Park, C. M. The miR172 target TOE3 represses *AGAMOUS* expression during *Arabidopsis* floral patterning. *Plant Science* **215-216**, 29-38 (2014).
