# Supplementary material for: Cytokinin regulates vegetative phase change in Arabidopsis thaliana through the miR172/TOE1-TOE2 module
Source: Nat Commun. 2021 Oct 5;12:5816. doi: 10.1038/s41467-021-26088-z (PMC8492644; doi:10.1038/s41467-021-26088-z)
Supplement: Supplementary file 3 — Reporting Summary [file 41467_2021_26088_MOESM3_ESM.pdf]

Corresponding author(s): Thomas Schmülling
Last updated by author(s): Sep 1, 2021

Reporting Summary

Nature Portfolio wishes to improve the reproducibility of the work that we publish. This form provides structure for consistency and transparency in reporting. For further information on Nature Portfolio policies, see our Editorial Policies and the Editorial Policy Checklist.

Statistics

For all statistical analyses, confirm that the following items are present in the figure legend, table legend, main text, or Methods section.

n/a	Confirmed	
☐	☒	The exact sample size (*n*) for each experimental group/condition, given as a discrete number and unit of measurement
☐	☒	A statement on whether measurements were taken from distinct samples or whether the same sample was measured repeatedly
☐	☒	The statistical test(s) used AND whether they are one- or two-sided<br>*Only common tests should be described solely by name; describe more complex techniques in the Methods section.*
☒	☐	A description of all covariates tested
☐	☒	A description of any assumptions or corrections, such as tests of normality and adjustment for multiple comparisons
☐	☒	A full description of the statistical parameters including central tendency (e.g. means) or other basic estimates (e.g. regression coefficient) AND variation (e.g. standard deviation) or associated estimates of uncertainty (e.g. confidence intervals)
☐	☒	For null hypothesis testing, the test statistic (e.g. *F*, *t*, *r*) with confidence intervals, effect sizes, degrees of freedom and *P* value noted<br>*Give P values as exact values whenever suitable.*
☒	☐	For Bayesian analysis, information on the choice of priors and Markov chain Monte Carlo settings
☒	☐	For hierarchical and complex designs, identification of the appropriate level for tests and full reporting of outcomes
☒	☐	Estimates of effect sizes (e.g. Cohen's *d*, Pearson's *r*), indicating how they were calculated

Our web collection on statistics for biologists contains articles on many of the points above.

Software and code

Policy information about availability of computer code

Data collection	qRT-PCR experiments were performed using Bio-Rad CFX96 Real-Time System and Bio-Rad CFX Manager (v3.1.1517.0823).<br>Agarose gel pictures shown in Fig. S11b were taken using the GenoPlex gel documentation system and GenoCapture v7.12.09.0 (VWR).
Data analysis	For statistical analysis, Prism v8.4.3 (GraphPad) was used.<br>Box plots and all bar charts (except for Figs. S4 and S9) were generated using RStudio v1.3.1056 with the ggplot2 (version 3.3.2) package.<br>All other graphs were generated using Microsoft Excel 2019.<br>qRT-PCR data analysis was carried out according to Vandesompele et al. (2002) and Morcuende et al. (2007).

For manuscripts utilizing custom algorithms or software that are central to the research but not yet described in published literature, software must be made available to editors and reviewers. We strongly encourage code deposition in a community repository (e.g. GitHub). See the Nature Portfolio guidelines for submitting code & software for further information.

Data

Policy information about availability of data

All manuscripts must include a data availability statement. This statement should provide the following information, where applicable:

- Accession codes, unique identifiers, or web links for publicly available datasets
- A description of any restrictions on data availability
- For clinical datasets or third party data, please ensure that the statement adheres to our policy

Source data and statistical information are provided with this paper.

Field-specific reporting

Please select the one below that is the best fit for your research. If you are not sure, read the appropriate sections before making your selection.

☒ Life sciences ☐ Behavioural & social sciences ☐ Ecological, evolutionary & environmental sciences

For a reference copy of the document with all sections, see nature.com/documents/nr-reporting-summary-flat.pdf

Life sciences study design

All studies must disclose on these points even when the disclosure is negative.

Sample size	No statistical method was used to predetermine sample size. The sample sizes are standard for this type of experiments and the reproducibility between independent experiments indicates sample sizes to be sufficient.
Data exclusions	No data were purposely excluded. However, individual plants that died or developed abnormal phenotypes during the experiments were not examined and therefore excluded from the analysis. Single samples during qRT-PCR analyses did not yield a fluorescence signal, but respective runs were not repeated if other samples were fine (Fig. 4a, SPL15 at time point 0; Fig. 5c, miR172 (Treated) at time point 0.5; Fig. S3a, ARR6 (Mock) at time point 0.5, ARR9 (Mock) at time point 0.5; Fig. S3c, MIR156C (Mock) at time point 2; Fig. S6, ARR6 at time point 0; Fig. S7c, MIR172A CKX1ox at 7 dag, ckx3,4,5,6 at 14 dag, Col-0 at 21 dag).
Replication	All experiments were repeated at least two times with similar results, with the exception of the analysis of transgenic T1 plants used in Figs. 5d, S8 and S9 which was only performed once.
Randomization	Samples were grown randomly placed within the same growth chamber to avoid positional effects, and allocated to groups based on genotype and treatment.
Blinding	The researchers were not blinded during this study, which would have been difficult due to the obvious phenotypes of the genetic material. Most of the results are quantitative, or not subjective.

Reporting for specific materials, systems and methods

We require information from authors about some types of materials, experimental systems and methods used in many studies. Here, indicate whether each material, system or method listed is relevant to your study. If you are not sure if a list item applies to your research, read the appropriate section before selecting a response.

Materials & experimental systems

n/a	Involved in the study	
☒	☐	Antibodies
☒	☐	Eukaryotic cell lines
☒	☐	Palaeontology and archaeology
☒	☐	Animals and other organisms
☒	☐	Human research participants
☒	☐	Clinical data
☒	☐	Dual use research of concern

Methods

n/a	Involved in the study	
☒	☐	ChIP-seq
☒	☐	Flow cytometry
☒	☐	MRI-based neuroimaging
